# The expression, immune infiltration, prognosis, and experimental validation of OSBPL family genes in liver cancer

**DOI:** 10.1186/s12885-023-10713-9

**Published:** 2023-03-14

**Authors:** Kunpeng Tian, Yongling Ying, Jingjing Huang, Hao Wu, Chengyue Wei, Liang Li, Longjun Chen, Lichuan Wu

**Affiliations:** 1 School of Medicine, Guangxi University, Nanning, Guangxi 530004 China; 2 Department of Spleen and Stomach Liver Diseases, The First Affiliated Hospital of Guangxi University of Traditional Chinese Medicine, Nanning, Guangxi 530200 China; 3 Guangxi Key Laboratory of Translational Medicine of Integrated Traditional Chinese and Western Medicine, Nanning, Guangxi 530200 China; 4 Guangxi Key Laboratory of Molecular Biology of Preventive Medicine of Traditional Chinese Medicine, Nanning, Guangxi 530200 China

**Keywords:** OSBPL family, Liver cancer, Expression, Immune infiltration, Prognosis, Experimental validation, Bioinformatics

## Abstract

**Background:**

Liver cancer is the third most deadly malignant tumor in the world with poor prognosis and lacks early diagnostic markers. It is urgent need to explore new biomarkers and prognostic factors. The oxysterol-binding protein-like family proteins (OSBPLs) are essential mediators of lipid transportation and cholesterol balancing which has been reported to participate in cancer progression. So far, the expression, immune infiltration, and prognosis of OSBPLs have not been elucidated in liver cancer.

**Methods:**

The differential expressions of OSBPLs between liver tumor and normal tissues were assessed by analyzing RNA-seq data from TCGA and protein data from CPTAC, respectively. Subsequently, genetic variations, potential functional enrichment analysis, and immune cell infiltration were analyzed. Further, the prognostic effects of OSBPLs were identified via constructing lasso models and performing receiver operating characteristic curve (ROC) analysis. Moreover, 10 local liver cancer specimens were involved to validate the expression of OSBPL3 via immunohistochemistry (IHC) assay. Finally, CCK-8, cell cycle, apoptosis, transwell assays, real time qPCR (RT-qPCR), and western blot assays were conducted to explore the function of OSBPL3 in liver cancer cells.

**Results:**

The mRNA of OSBPL2, OSBPL3, and OSBPL8 were highly expressed while OSBPL6 was lowly expressed in liver cancer samples compared with normal samples. As to the protein expression, OSBPL2 and OSBPL3 were significantly elevated and OSBPL5, OSBPL6, OSBPL9, OSBPL10, OSBPL11 were downregulated in tumor samples. A positive correlation was found between copy number variations (CNV) and the expression of OSBPL2, OSBPL8, OSBPL9, OSBPL11, while DNA methylation was negatively associated with the expressions of OSBPLs. Of these, CNV amplification mainly contributed to the overexpression of OSBPL2 and DNA methylation may be responsible for the high expression of OSBPL3. Interestingly, OSBPL3, OSBPL5, SOBPL7, and OSBPL10 were significantly positively correlated with immune infiltration. Notably, OSBPL3 was identified correlated to overall survival (OS) and disease specific survival (DSS) in liver cancer. Functionally, knocking down OSBPL3 reduced liver cancer cell viability, induced a G2/M cell cycle arrest, promoted apoptosis, and restrained cell migration.

**Conclusion:**

In aggregate, we reported a heretofore undescribed role of OSBPLs in liver cancer by analyzing multi-omics data. Importantly, we identified OSBPL3 was overexpressed in liver tumor compared with normal and its high expression was correlated with poor OS and DSS. Inhibition of OSBPL3 resulted in a pronounced decrease in cell proliferation and migration.

**Supplementary Information:**

The online version contains supplementary material available at 10.1186/s12885-023-10713-9.

## Introduction

Liver cancer is the third cause of cancer related death with an estimate of 830,180 deaths in 2020[1]. Risk factors of liver cancer include virus infection (hepatitis B and hepatitis C), cirrhosis, fatty liver disease, smoking, obesity, and so [2]. Hepatocellular carcinoma (HCC), intrahepatic cholangiocarcinoma (ICC), and HCC-ICC mixed type are the main types of liver cancer. Currently, the main clinical treatments for liver cancer include surgery, chemotherapy, radiotherapy, targeted therapy, and [3]. Compared to other treatments, surgery represents as the best treatment for patients with early liver cancer. However, since liver cancer patients have no obvious symptoms in the early stage and there is lacking of diagnostic marker, most of the patients were diagnosed with an advanced stage, missing the optimal timing for [4]. Alpha-fetoprotein (AFP) is the only clinical applied biomarker for diagnosis of early liver cancer which lacks accuracy as 32%~59% of liver cancer patients display normal AFP [5]. Meanwhile, liver cancer patients show a poor prognosis and the five-year survival rate is only about 30 − 50% with an average survival period of 3–6 [6]. Despite progress in liver cancer diagnostic and therapeutic techniques has been made, the outlook for liver cancer patients is still poor. Thus, a reliable predictive biomarker is urgent need to identify patients with high risk of liver cancer and to direct more personalized treatment. .

The oxysterol-binding protein-like (OSBPL) protein family includes nine members namely OSBPL2, OSBPL3, OSBPL5, OSBPL6, OSBPL7, OSBPL8, OSBPL9, OSBPL10, and OSBPL11[7]. The OSBPL family proteins (OSBPLs) locate at the membrane to exchange molecules or signals between organelles and function as a necessary transporter of [7]. In addition to lipid transport, the OSBPLs are involved in the regulation of the actin cytoskeleton, cell polarity, and cell [8]. Recently, the role of OSBPLs in cancer development has been gradually revealed. For instance, RNAi knockdown of OSBPL2 led to growth reduction in gastric [9]. Upregulation of OSBPL3 could promote colorectal cancer [10]. The activation of LMCD1-AS1/miR-562b-3p/OSBPL5 axis increased cell proliferation, migration, and invasion in non-small cell lung [11]. OSBPL7 and OSBPL8 were identified to be overexpressed in [12]. Mutations of OSBPL10 was identified as driver gene of breast [13]. Overexpression of OSBPL11 could reverse miR-7-5p induced cell proliferation inhibition in [14]. However, relatively little is known about the role of OSBPLs in liver cancer progression.

**Fig. 1:**
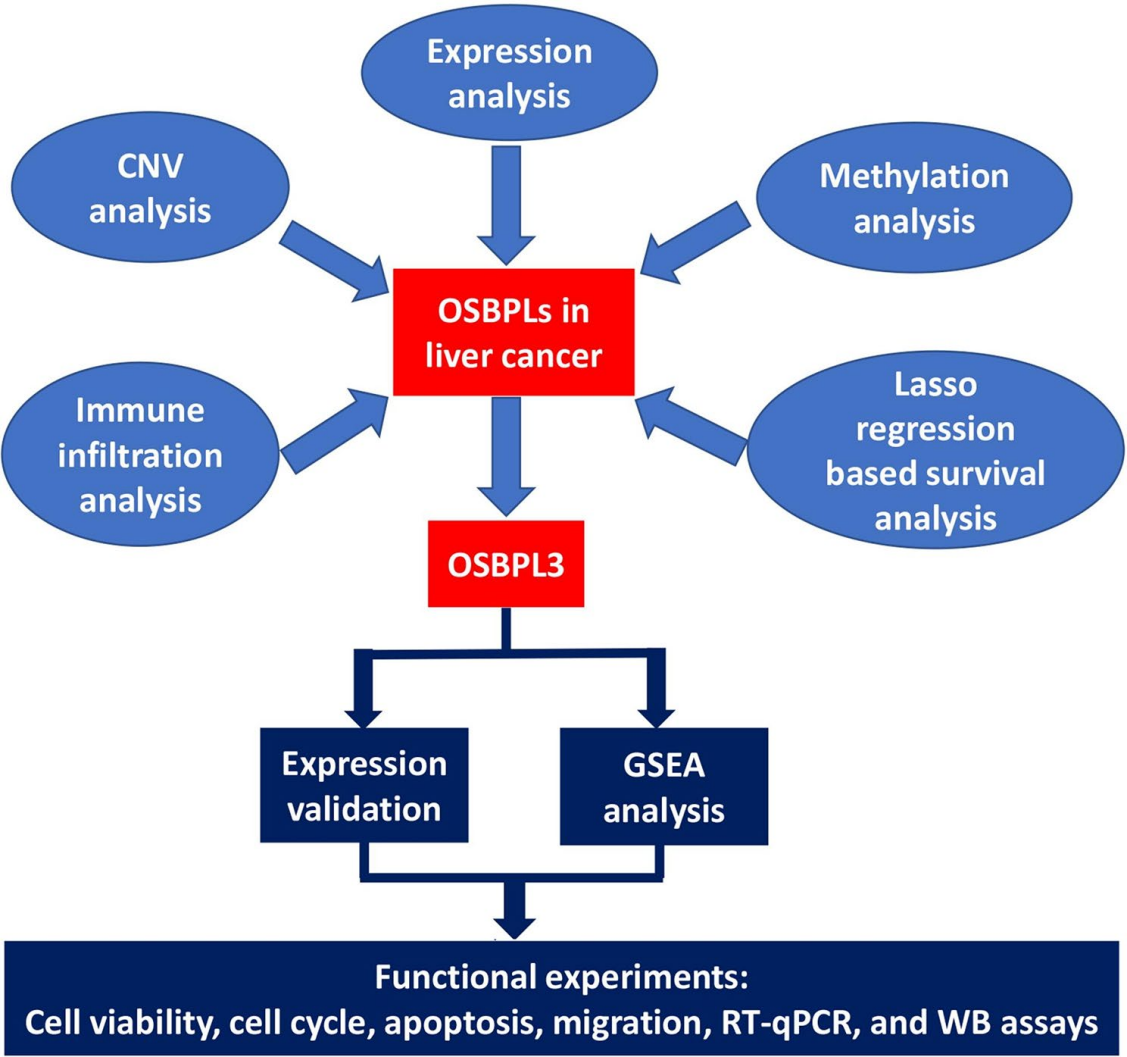
Flow chart of the present design

In the present study, the transcriptome, genomic, epigenetic, immune infiltration, and prognosis of OSBPLs were explored in liver cancer. Immunohistochemistry (IHC) was applied to validate the expression of OSBPL3 in liver cancer. CCK-8, cell cycle, apoptosis, transwell assays, real time qPCR (RT-qPCR), and western blot assays were conducted to investigate the biologic functions of OSBPL3 in liver cancer cells (Fig. 1). Our findings will provide additional information on the significance of OSBPLs, especially OSBPL3, in liver cancer progression.

## Materials and methods

### Specimen collection

Liver cancer tissues and matched normal adjacent tissues (≥ 2 cm away from cancer) were obtained from the First Affiliated Hospital of Guangxi University of Chinese Medicine with written informed consent of patients. Tumor cells account for approximately 80% of liver cancer tissue. This study was approved by Ethics Committee of the First Affiliated Hospital of Guangxi University of Chinese Medicine (2020-009-02). All experiments in this study were conducted in accordance with the guidelines in the Declaration of Helsinki.

### Cell culture and siRNA transfection

The liver cancer cells (PLC/PRF/5, SMMC-7721, HepG2, MHCC-97 H, Bel-7402, and SK-Hep-1) were purchased from NANJING COBIOER BIOSCIENCES CO., LTD and cultured in Dulbecco’s Modified Eagle Medium (DMEM) supplemented with 10% fetal bovine serum (FBS) and 1% penicillin-streptomycin. PLC/PRF/5 Cells were transfected with negative control (NC) or small interfering RNA of OSBPL3 (siOSBPL3) (sense: GCUUUCUAAUGAAAGUAGATT, and anti-sense: UCUACUUUCAUUAGAAAGCTG) by using lipofectamine 3000 (Invitrogen) in 12 well plate (0.66 µg per well). Twenty-four hours later, the cells were harvested for cell viability, cell cycle, transwell assays, and western blot assays.

### Data acquisition, processing, and differentially expressed genes (DEGs) analysis

RNA seq data of liver cancer (160 normal and 371 tumor) preprocessed by python package “toil” were retrieved from UCSC XENA database (https://xenabrowser.net/datapages/?host=https%3 A%2 F%2Ftoil.xenahubs.net) and analyzed via applying R package “stats” and “car”. The microarray data (GSE121248, GSE25097, GSE45436, and GSE62232) was downloaded from GEO database (https://www.ncbi.nlm.nih.gov/geo/) and processed with R package “RMA”. Subsequently, data were processed by running R package “limma” to obtain DEGs. Genes with a criterion of p < 0.05 and |Fold change| >2 were considered as DEGs between normal and tumor samples.

### Protein expression analysis

The protein expressions of OSBPLs between liver tumor and normal tissues were determined via using CPTAC database (http://ualcan.path.uab.edu/analysis-prot.html)[15]. Briefly, liver hepatocellular carcinoma in the CPTAC dataset was selected and gene ID (official gene symbol) were imputed. p < 0.05 was considered as statistical significance.

### Analysis of copy number variations (CNV) and methylation

Online database of Gene Set Cancer Analysis (GSCA) (http://bioinfo.life.hust.edu.cn/GSCA/#/) which assembles various tumor genome data including CNV, mutation, methylation and immune [16] was applied to evaluate the CNV and methylation levels of OSBPLs. The Expression module and Mutation module was selected to perform CNV analysis and methylation analysis, respectively. In brief, gene IDs (official gene symbol) were imputed and liver hepatocellular carcinoma was chosen which includes 374 tumor and 50 normal samples.

### Immune infiltration analysis

The correlation between expression of OSBPLs and immune infiltrates were determined using TIMER database (https://cistrome.shinyapps.io/timer/)[17]. The Gene module was applied to detect the correlation between OSBPLs expression and immune cells infiltration. Briefly, gene IDs were imputed into the Gene Symbol box and in the Cancer Types box, liver hepatocellular carcinoma was selected. While in the Immune Infiltrates box, B cell, CD8 + T cell, CD4 + T cell, macrophage, neutrophil, and dendritic cell were chosen.

### Lasso regression analysis

RNA-seq data (level 3) of liver cancer filtered with normal control samples was downloaded from TCGA. First, the expression data (Fragments Per Kilobase per Million, FPKM) of OSBPLs was converted to Transcripts Per kilobase per Million (TPM) following a log2 transformation. Then, the expression matrix of OSBPLs with clinical information was processed via running R packages “glmnet” and “survival” to construct a risk score system.

### Survival analysis

The median risk score of the combining of OSBPL3 and OSBPL10 was used to divide group into high and low risk groups for OS. Also, the median risk score of the combining of OSBPL3 and OSBPL6 was used to divide group into high and low risk groups for DSS. Then, the Kaplan-Meier (K-M) survival analysis was conducted via running R package “survminer”. The time-related receiver operating characteristic curve (ROC) was performed to assess the OS predictive ability of OSBPL3 in liver cancer by running R package “pROC”.

### Gene Set Enrichment Analysis (GSEA)

RNA-seq data (level 3) of liver cancer was downloaded from TCGA via running R package “TCGAbiolinks”. Then DEGs between OSBPL3 highly and lowly expressed groups were identified via running R package “DESeq2” with a criterion of p < 0.05 and |Fold change| >2. Meanwhile, expression correlation analysis of OSBPL3 was conducted via running R package “stat”. Subsequently, up-regulated DEGs with a correlation value r > 0.5 were involved in the GSEA analysis by running R package “clusterProfiler”.

### Immunohistochemistry (IHC) assay

A total of 10 liver cancer samples with matched normal adjacent tissues were involved to validate the expression of OSBPL3 by IHC assays as previously [18]. Briefly, the paraffin-embedded tissues were deparaffinized and rehydrated by xylene and graded alcohol relatively. Then, antigen was retrieved by heat induced epitope retrieval and citrate buffer. Subsequently, the sections were blocked with 5% BSA and incubated with OSBPL3 antibody (Proteintech, China, Cat No: 12417-1-AP) at 4 °C overnight. After 24 h, the sections were washed and incubated with secondary antibody for 90 min at 37 ℃, following a detection by 3, 3 ′ -diaminobenzidine and staining by hematoxylin. At last, the sections were dehydrated, cleared, mounted and scored with a calculation formula, IRS (0–12) = RP (0–4) × SI (0–3), in which RP and SI refers to the percentage of staining-positive cells and staining intensity, respectively.

### Cell viability assay

PLC/PRF/5 Cells transfected with NC or siOSBPL3 were plated in 96-well plates with 5000 cells per well. CCK-8 was added after indicated time (24, 48, and 72 h)and incubated for additional 2 h. Then, the plates were assayed by testing the absorbance at 450 and 600 nm.

### Cell cycle and apoptosis assays

For cell cycle analysis, PLC/PRF/5 cells were transfected with NC or siOSBPL3 in 12-well plate. After 24 h, cells were harvested and cell cycle was determined by using a cell cycle detection kit (KeyGen BioTECH, Cat: KGA512) according to the kit instruction. For apoptosis assay, PLC/PRF/5 cells were transfected with NC or siOSBPL3 for 48 h. Then, cells were collected and cell apoptosis was detected using an Annexin V/7-AAD Apoptosis Detection kit (BioLegend (USA), Cat: 640,922) according to the kit instruction.

### RNA extraction and real-time qPCR (RT-qPCR)

RNA extraction and real-time qPCR were conducted as reported [19]. Briefly, RNA was extracted by using TRIzol reagent (Life Technologies, Scotland, UK) according to the protocol. Subsequently, a total of 1 µg RNA was used to perform reverse transcription by using HiScript II Q Select RT SuperMix (Vazyme, Nanjing, China). Finally, RT-qPCR was performed using SYBR Select Master Mix (Applied Biosystems, catalog no. 4,472,908) with the ABI7300 system (Applied Biosystems, Foster City, CA, USA). The primer for OSBPL3, sense (5’-3’): GCUUUCUAAUGAAAGUAGAtt, antisense (5’-3’):UCUACUUUCAUUAGAAAGCtg.

### Western blot assay

PLC/PRF/5 Cells transfected with NC or siOSBPL3 for 48 h were harvested and lysed in RIPA buffer. Then, cell lysates were centrifuged at 12,000 x g for 10 min at 4 °C to obtain proteins. Next, proteins were subjected to 8–15% SDS-PAGE, electrophoresed, and transferred on to a PVDF membrane following blocking with 5% non-fat milk dissolved in trisbuffered saline. After washing with trisbuffered saline, the membrane was incubated with indicated primary and secondary antibodies. Finally, proteins were detected using the Luminescent Image Analyser LSA 4000 (GE, Fairfield, CO, USA). The primary antibodies involved in this study include Bax (Cell Signaling Technology, USA, #41,162), PARP (Cell Signaling Technology, USA, #9532), Caspase-3 (Cell Signaling Technology, USA, #9662), and β-actin (Cell Signaling Technology, USA, #4970).

### Cell migration assays

Transwell inserts (LABSELECT, Cat: 14,341) were pretreated with serum-free medium for 1 h. Cells transfected with NC or siOSBPL3 for 24 h were plated into each insert (the upper chamber). Medium with 10% of FBS was loaded into the bottom chamber. Twenty-four hours later, cells in the upper surface of the insert membrane were removed, while cells in the lower surface were fixed with methanol, stained with crystal violet, and counted by microscopy.

### Statistical analysis

Differences between data groups were evaluated for significance using Student t-test. P values < 0.05 were considered statistically significant. All experiments were repeated at least three times and data are presented as the mean ± SD unless noted otherwise. *, p < 0.05; **, p < 0.01; ***, p < 0.001.

## Results

### The mRNA and protein expressions of OSBPLs in liver cancer

**Fig. 2:**
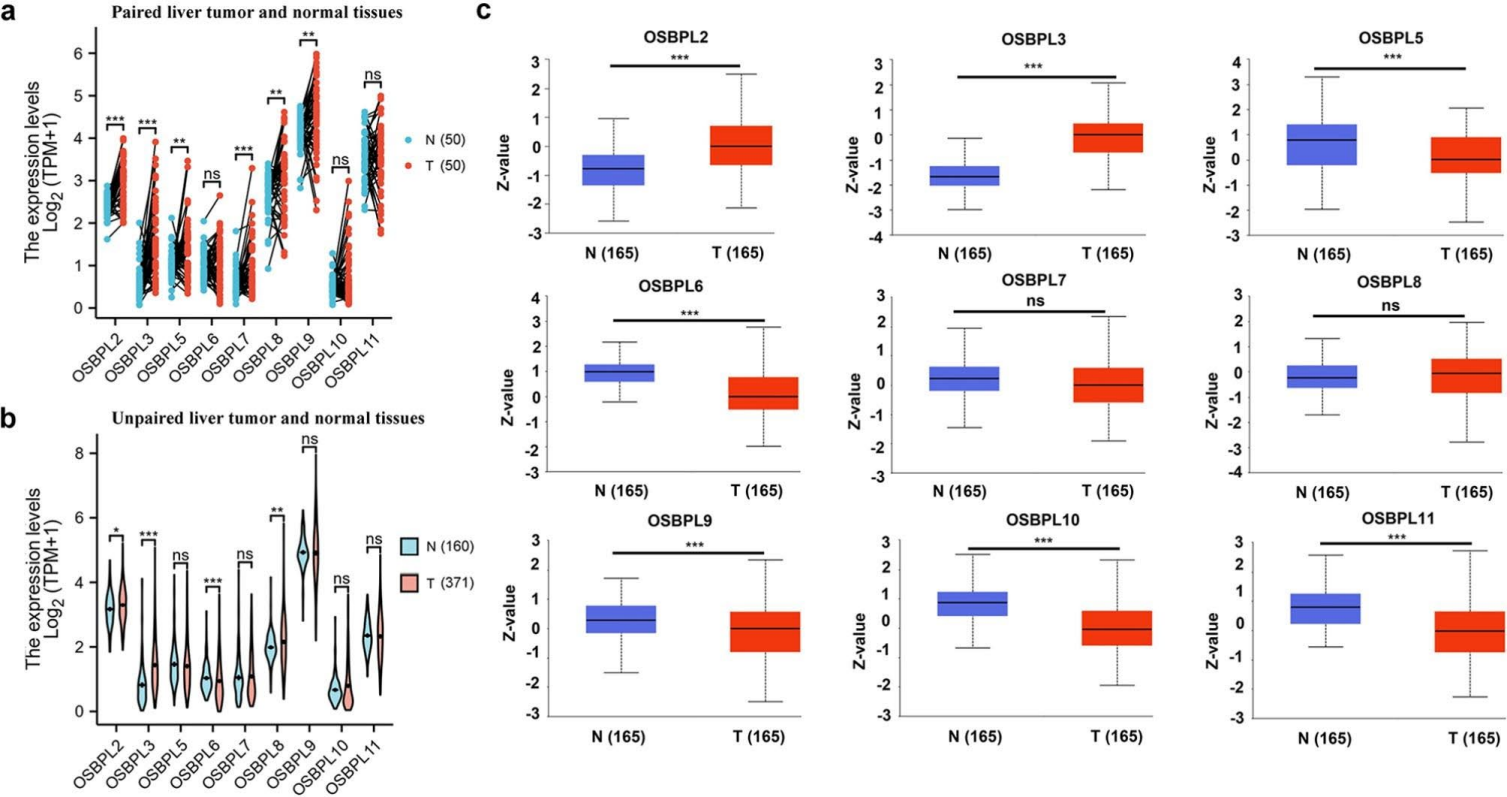
**The mRNA and protein expressions of OSBPLs in liver tumor and normal tissues.** (a) The mRNA expressions of OSBPLs in paired liver tumor and normal tissues from TCGA. (b) The mRNA expressions of OSBPLs in unpaired liver tumor and normal tissues from TCGA and GTEx. (c) The protein expressions of OSBPLs in liver tumor and normal tissues from CPTAC database

The mRNA expressions of OSBPLs in liver tumor and matched normal tissues (50 pairs) were examined first by analyzing data from TCGA which demonstrated that OSBPL2, OSBPL3, OSBPL5, OSBPL7, OSBPL8, and OSBPL9 were upregulated (Fig. 2a). To further confirm their expression in a large cohort, 160 normal and 371 tumor samples from TCGA and GTEx were employed. The results revealed that OSBPL2, OSBPL3, and OSBPL8 were overexpressed while OSBPL6 was downregulated in liver tumor (Fig. 2b). Subsequently, the protein expressions of OSBPLs in liver tumor and normal tissues were determined using CPTAC database. The results demonstrated that the protein expressions of OSBPL2 and OSBPL3 were elevated while OSBPL5, OSBPL6, OSBPL9, OSBPL10, and OSBPL11 were lowly expressed in tumor samples (Fig. 2c). Taken together, these results imply that OSBPL2, OSBPL3, and OSBPL6 were potentially involved in liver cancer progression.

### Association between CNV/methylation and mRNA expressions of OSBPLs in liver cancer

**Fig. 3:**
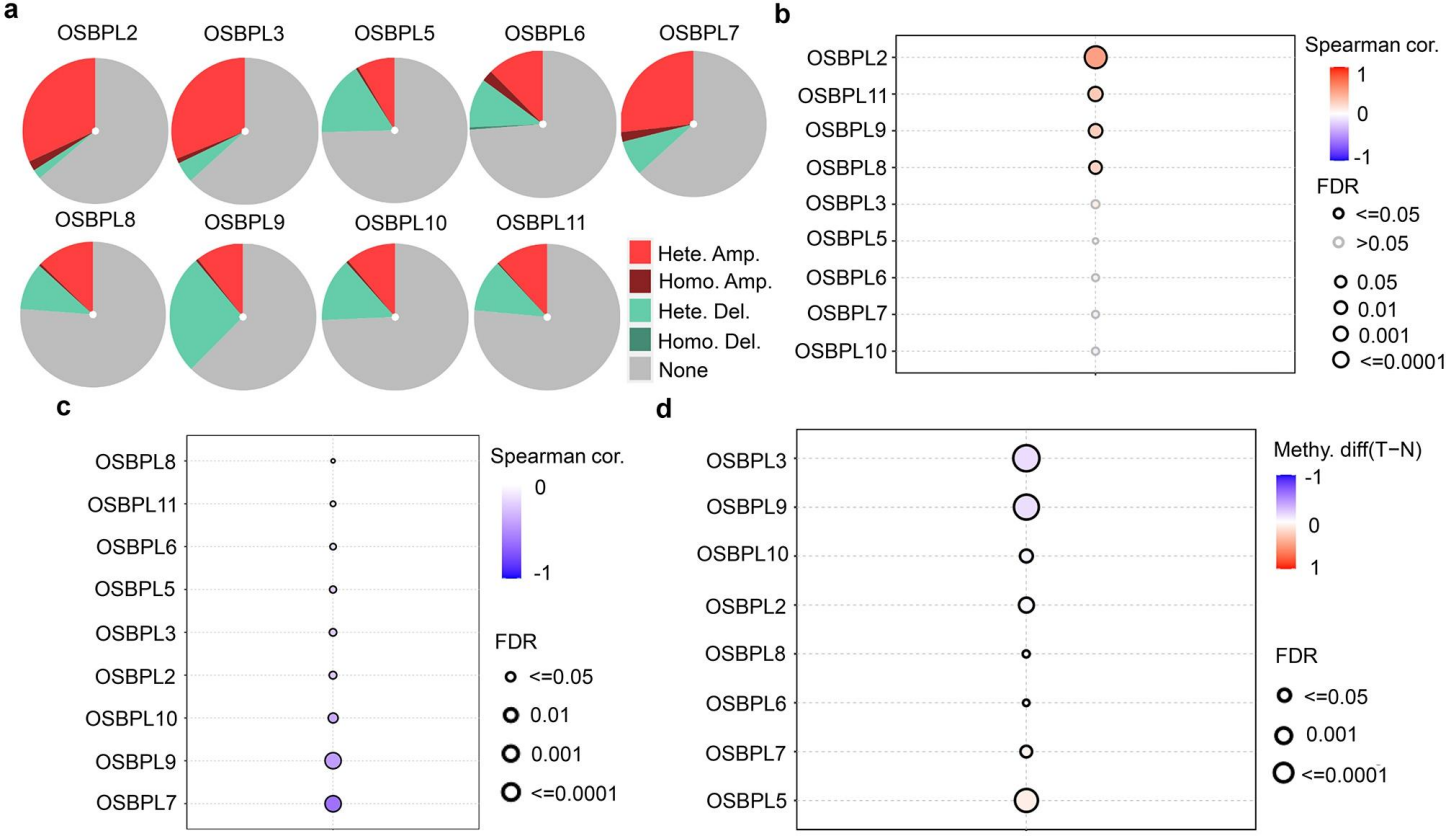
**CNV and DNA methylation differences of OSBPLs in liver cancer.** (a) CNV of OSBPLs in liver cancer. (b) Correlations between CNV and OSBPLs expressions. (c) Correlations between DNA methylation and OSBPLs expressions. (d) DNA Methylation of OSBPLs between liver tumor and normal tissues. Hete. Amp: heterozygous amplification; Homo. Amp: homozygous amplification; Hete. Del: heterozygous deletion; Homo. Del: homozygous deletion; Methy. diff (T-N): methylation difference between tumor and normal tissues

To better understand the abnormal expressions of OSBPLs in liver cancer, we first explored the contribution of CNV to OSBPLs expression. Different degrees of CNV were found which displayed that amplification was the major CNV pattern for OSBPL2, OSBPL3, and OSBPL7. While deletion appeared as the major CNV for OSBPL5 and OSBPL9 (Fig. 3a). A positive correlation was found between the expression of OSBPL2, OSBPL8, OSBPL9, OSBPL11 and CNV (Fig. 3b). Thereafter, we characterized the association between DNA methylation and OSBPLs expression. The results exhibited that methylation level was negatively correlated with the expression of OSBPLs (Fig. 3c). Furthermore, DNA methylation difference between tumor and normal were tested. We observed a significantly lower expression of DNA methylation for OSBPL3 and OSBOL9 while a remarkably higher expression of DNA methylation for OSBPL5 and OSBPL7 in liver tumor compared with normal tissues (Fig. 3d). In aggregate, these results suggest that CNV amplification mainly contributed to the overexpression of OSBPL2 while DNA methylation may be responsible for the high expression of OSBPL3.

### Relationship between the expression of OSBPLs and immune infiltration in liver cancer

**Fig. 4:**
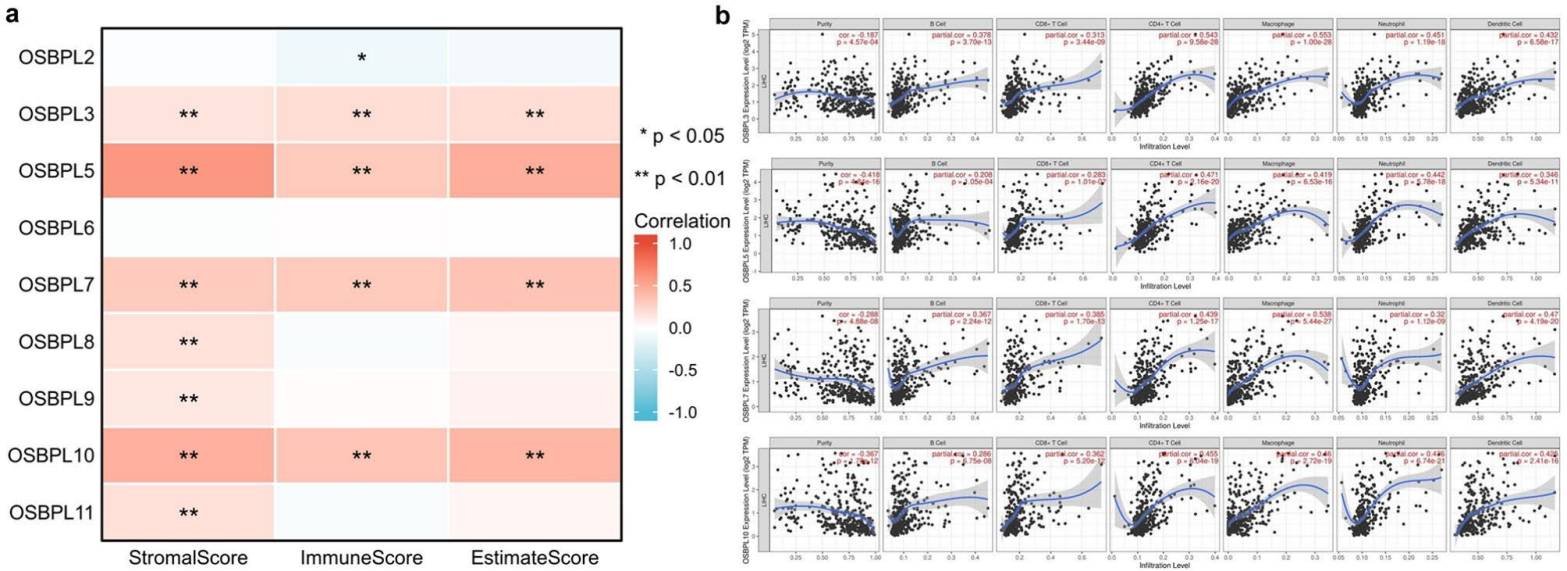
**Correlations between OSBPLs expression and immune infiltration.** (a) The immune scores of OSBPLs in liver cancer using three different strategies including StromalScore, ImmuneScore, and EstimateScore. (b) Correlation analysis between OSBPLs mRNA expression and infiltration of six major immune cells

Tumor-infiltrating lymphocytes are an independent predictor of patient survival in cancers. We asked whether OSBPPLs expression was associated with immune infiltration levels in liver cancer. We first calculated the immune score of OSBPLs using three different strategies including StromalScore, ImmuneScore, and EstimateScore which exhibited that OSBPL3, OSBPL5, SOBPL7, and OSBPL10 were significantly positively correlated with immune infiltration (Fig. 4a). Afterwards, we assessed the relation between expression of OSBPL3, OSBPL5, SOBPL7, OSBPL10 and infiltration of different immune cells types. The results displayed that the expressions of OSBPL3, OSBPL5, SOBPL7, and OSBPL10 were significantly correlated with tumor purity, B cell, CD8 + T cells, CD4 + T cells, macrophages, neutrophils, and dendritic cells infiltration levels (Fig. 4b).

### Survival model construction and analysis

**Fig. 5:**
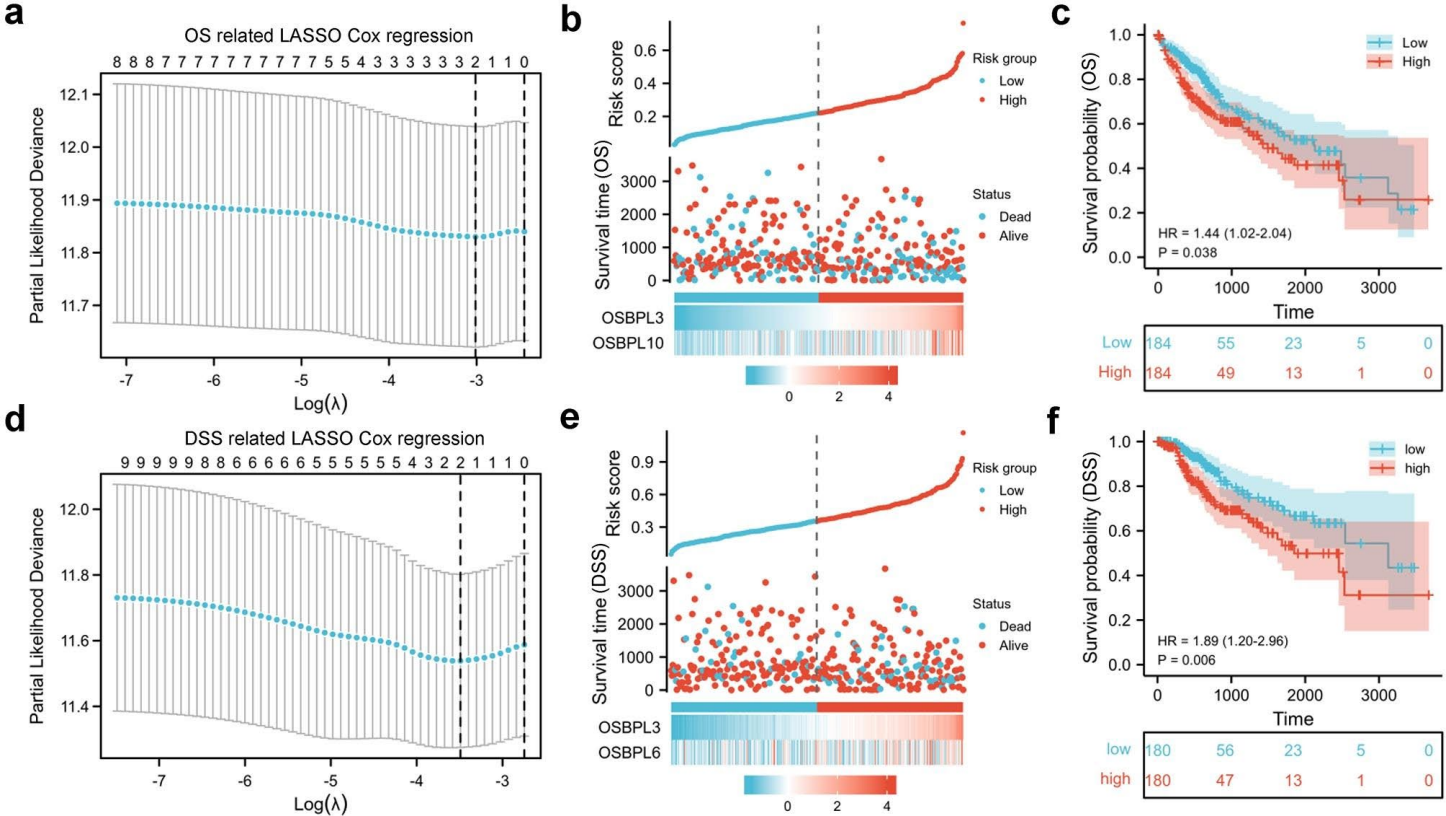
**Construction of OSBPLs-related risk score.** (a) The OS related LASSO Cox regression model was constructed from OSBPLs. (b) The distribution, the OS status of each sample, and the expression value of the two crucial genes. (c) The OS prognostic significance of gene signature containing OSBPL3 and OSBPL10. (d) The DSS related LASSO Cox regression model was constructed from OSBPLs. (e) The distribution, the DSS status of each sample, and the expression value of the two crucial genes. (f) The DSS prognostic significance of gene signature containing OSBPL3 and OSBPL6.

**Fig. 6:**
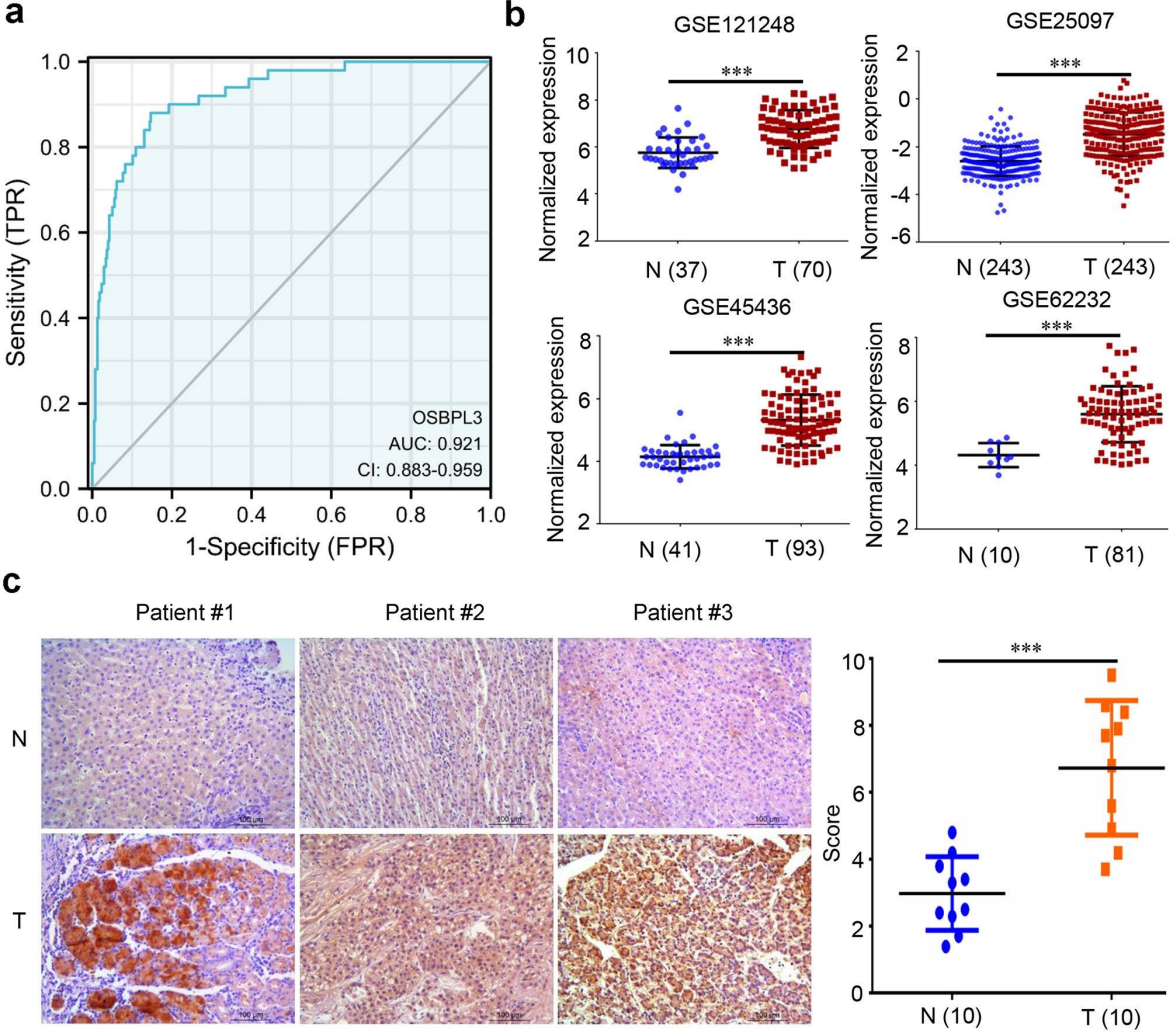
**Expression validation of OSBPL3 in liver cancer.** (a) ROC analysis of OSBPL3 using the TCGA LIHC dataset. (b) The mRNA expression validation of OSBPL3 in GEO datasets. (c) The protein expression validation of OSBPL3 in 10 local liver tumor and matched normal tissues

To assess the contribution of gene expression to OS, members of OSBPLs were then input into a LASSO regression model which generated two key genes, OSBPL3 and OSBPL10 (Fig. 5a-b). Based on the risk score, patients of liver cancer were classified into the high and low groups and patients in high group exhibited significantly shorter survival compared with patients in the low group (Fig. 5c). Furthermore, a LASSO regression model was also constructed to estimate the impacts of gene expression to DSS which exerted OSBPL3 and OSBPL6 as two crucial candidates (Fig. 5d-e). Patients were also divided into high and low risk score groups and the high risk group showed poor DSS compared with the low risk group (Fig. 5f). Notably, OSBPL3 appeared as the only gene related to both OS and DSS in these two lasso models, indicating that OSBPL3 was a prognostic factor of liver cancer. Then we performed ROC analysis to evaluate the diagnostic potency of OSBPL3 in liver cancer which demonstrated that OSBPL3 was a strong predictor (AUC = 0.921, CI = 0.883–0.959) (Fig. 6a).

### Expression validation of OSBPL3 in liver cancer

Given our findings that OSBPL3 is overexpressed and prognostic in liver cancer, we hypothesized that OSBPL3 may play a crucial role in liver cancer development. First, expression validation of OSBPL3 in liver cancer was conducted by analyzing four cohorts of GEO datasets which demonstrated that OSBPL3 mRNA was drastically increased in tumor samples (Fig. 6b). Moreover, the protein of OSBPL3 was also found highly expressed in 10 local liver tumor samples (Fig. 6c).

### GSEA analysis of OSBPL3 in liver cancer

**Fig. 7:**
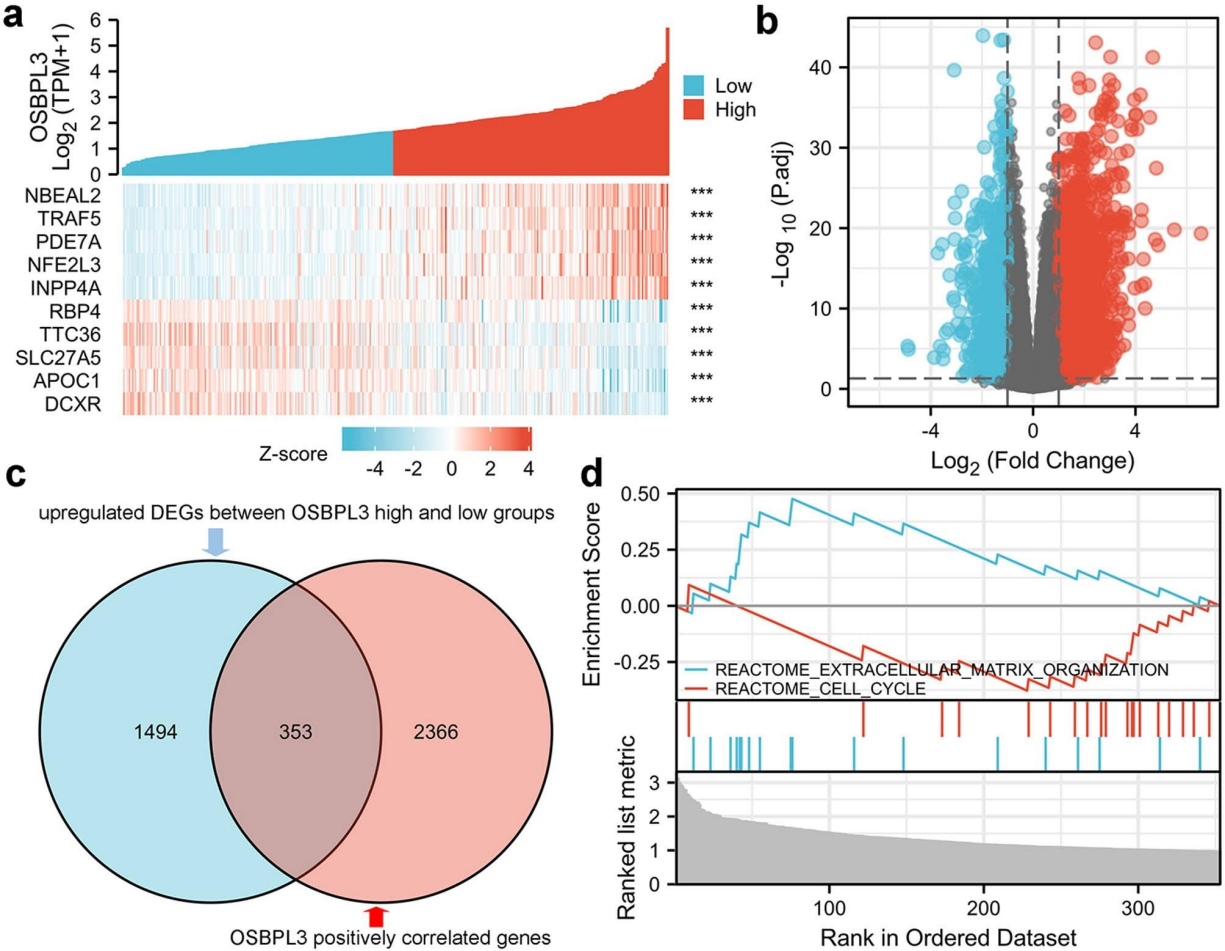
**GESA analysis of OSBPL3 functionally associated genes.** (a) Top 10 OSBPL3 expression correlated genes. (b) DEGs between OSBPL3 high and low expressed groups. (c) Intersection between OSBPL3 positively correlated genes and upregulated DEGs. (d) Cell cycle and extracellular matrix organization pathway were significantly enriched in the 353 intersected genes

To better understand the potential mechanism of OSBPL3 in liver cancer tumorigenesis, RNA-seq data from TCGA was used to perform gene expression correlation analysis and differentially expressed genes (DEGs) analysis. A total of 2986 genes with |r|>0.5 were identified including 2978 positively and 8 negatively OSBPL3 correlated genes (Fig. 7a). While 4441 DEGs were revealed including 3352 upregulated and 1089 downregulated genes (Fig. 7b). To further identify functionally associated genes of OSBPL3, intersection was made between upregulated DEGs and positively correlated genes which led to 353 intersected genes (Fig. 7c). Afterwards, these 353 genes were involved to conduct GSEA analysis which showed that the cell cycle and extracellular matrix organization pathway were significantly enriched (Fig. 7d).

### Functional analysis of OSBPL3 in liver cancer cell

**Fig. 8:**
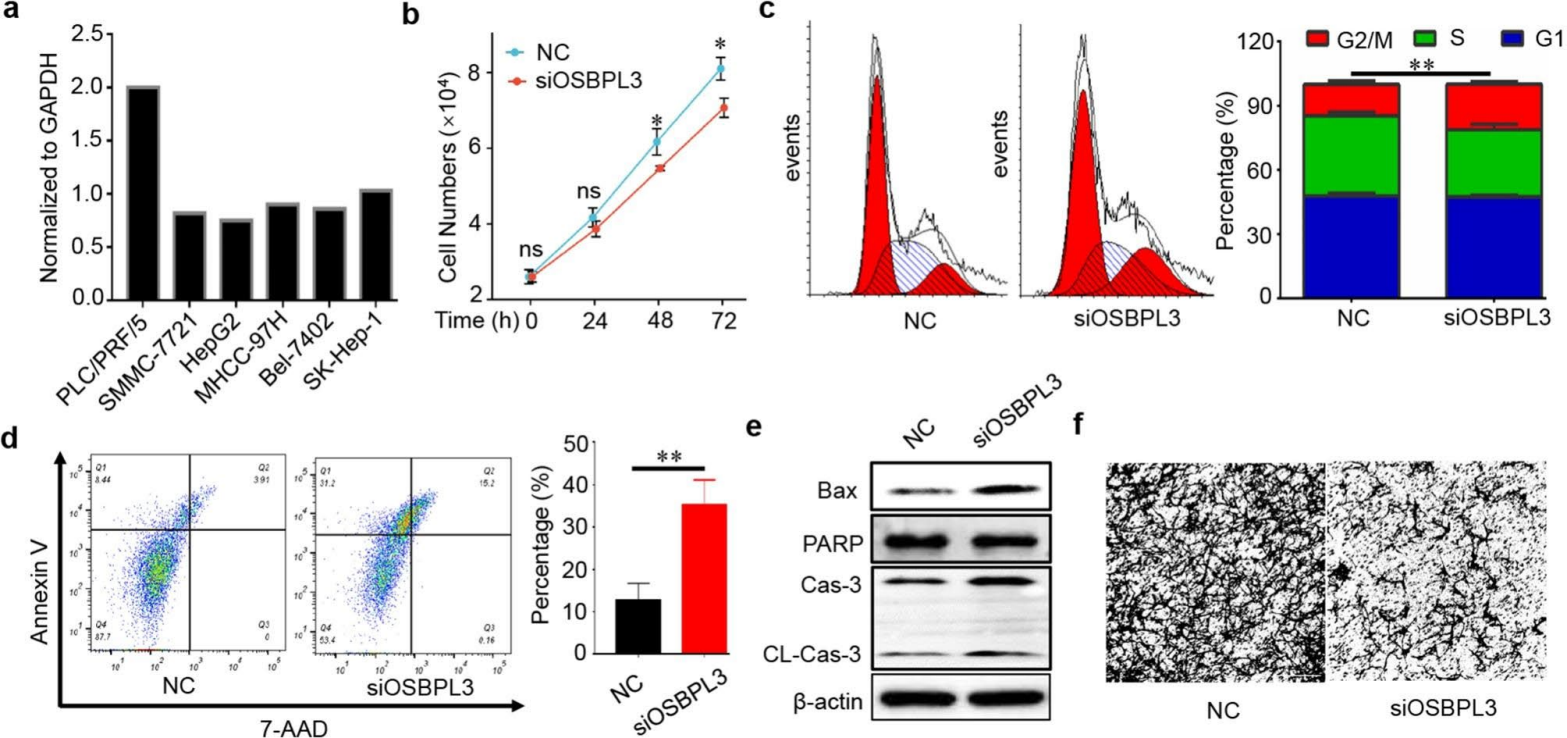
**Knockdown of OSBPL3 restrained cell proliferation and migration.** (a) The mRNA expression of OSBPL3 in six liver cancer cell lines. (b) Knockdown of OSBPL3 significantly reduced PLC/PRF/5 cell viability. (c) Knockdown of OSBPL3 after 24 h induced a G2/M cell cycle arrest in PLC/PRF/5 cell. (d) Knockdown of OSBPL3 after 48 h promoted PLC/PRF/5 cell apoptosis. (e) Knockdown of OSBPL3 after 48 h activated the pro-apoptotic proteins. (f) Knockdown of OSBPL3 suppressed PLC/PRF/5 cell migration

Deregulated cell cycle and extracellular matrix remodeling are important hallmarks of [20]. Given the data of GSEA, we sought to determine the role of OSBPL3 in the regulation of cell proliferation and migration. Initially, the mRNA expression of OSBPL3 in liver cancer cell lines were detected which displayed that OSBPL3 was highly expressed in PLC/PRF/5 compared to the other 5 liver cancer cell lines (Fig. 8a). Therefore, PLC/PRF/5 was selected for the further experiments. We discovered that knocking down OSBPL3 significantly inhibited cell proliferation and induced a G2/M cell cycle arrest (Fig. 8b-c). Interference of OSBPL3 also promoted a pronounced apoptosis (Fig. 8d). Pro-apoptotic proteins include Bax, PARP, and cleaved caspase-3 were activated upon OSBPL3 siRNA treatment (Fig. 8e). Additionally, cell migration was remarkably impeded in cells upon OSBPL3 siRNA transfection (Fig. 8f).

## Discussion

Abnormal lipid metabolism occurs frequently in tumor patients which could regulate gene expressions and modify signaling transduction [21]. Accumulation of cholesterol is considered as an obvious feature of tumor [22]. Enhanced cholesterol synthesis could activate Hedgehog signaling, leading to liver cancer stem cell self-renewal and [23]. Therefore, investigating cholesterol metabolism related genes could help to decipher the pathogenesis of liver cancer. The OSBPLs function as necessary lipid transports and maintain the balance of [24]. Additionally, OSBPLs act as important mediators of signaling transduction between intracellular and extracellular [25]. Presently, the role of OSBPLs in liver cancer has not been reported. In the present study, we found that the trend of mRNA expression of OSBPL2, OSBPL3, and OSBPL6 is consistent with that of protein expression (Fig. 2). Findings of genomic and epigenetic analysis demonstrated that CNV mainly contributed to the overexpression of OSBPL2 while DNA methylation accounts for the high expression of OSBPL3 (Fig. 3). These results infer a potential key role of OSBPL2 and OSBPL3 in liver cancer development.

Immune cells infiltration is gradually recognized as one of the main obstacles of cancer [26]. The role of OSBPLs in tumor immune infiltration has not been fully illustrated. By analyzing the correlation between gene expression and immune infiltration, we uncovered that OSBPL3, OSBPL5, SOBPL7, and OSBPL10 were significantly positively correlated with immune infiltration (Fig. 4). These results imply that these four genes might be involved in liver cancer microenvironment modeling.

In order to evaluate the prognostic of OSBPLs in liver cancer, OS and DSS related lasso models were constructed which exerted that OSBPL3 was a key prognostic factor to both OS and DSS (Fig. 5). The ROC analysis identified OSBPL3 was a diagnostic factor of liver cancer (Fig. 6a). Expression validation confirmed that OSBPL3 was significantly overexpressed in liver tumor compared with normal tissues (Fig. 6b-c). These results implied OSBPL3 might be involved in liver cancer progression. Therefore, we performed GSEA analysis to predict its biological function which showed that OSBPL3 might regulate cell proliferation and migration (Fig. 7). The subsequent functional experiments verified that knocking down OSBPL3 led to cell proliferation inhibition by inducing a G2/M cell cycle arrest and apoptosis and restrained cell migration (Fig. 8).These results implies that OSBPL3 might be a prognostic marker and regulator of liver cancer. To comprehensively elucidate the role of OSBPL3 in liver cancer progression, more data including in vivo experiments are needed.

## Conclusion

In conclusion, we assessed the expression, genomic, and epigenomic variation in liver cancer. Especially, we found OSBPL3 was overexpressed and a prognostic factor in liver cancer. Downregulating OSBPL3 induced a G2/M cell cycle arrest and apoptosis and suppressed cell migration.

## Electronic supplementary material

Below is the link to the electronic supplementary material.

Supplementary Material 1

## Data Availability

The RNA-seq datasets analysed during the current study are available in the UCSC repository: https://xenabrowser.net/datapages/?host=https%3 A%2 F%2Ftoil.xenahubs.net; The Gene expression datasets analysed during the current study are available in the GEO repository: https://www.ncbi.nlm.nih.gov/geo/. .

## Abbreviations

OSBPLs: The oxysterol-binding protein-like family genes
LIHC: Liver hepatocellular carcinoma
OS: Overall survival
DSS: Disease specific survival
GSCA: Gene Set Cancer Analysis
ROC: Receiver operating characteristic curve
CNV: Copy number variations
IHC: Immunohistochemistry
FPKM: Fragments Per Kilobase per Million
TPM: Transcripts Per kilobase per Million
GSEA: Gene Set Enrichment Analysis
DMEM: Dulbecco’s Modified Eagle Medium
FBS: Fetal bovine serum.
