# Supplementary material for: The expression, immune infiltration, prognosis, and experimental validation of OSBPL family genes in liver cancer
Source: BMC Cancer. 2023 Mar 14;23:244. doi: 10.1186/s12885-023-10713-9 (PMC10015719; doi:10.1186/s12885-023-10713-9)

First and Second

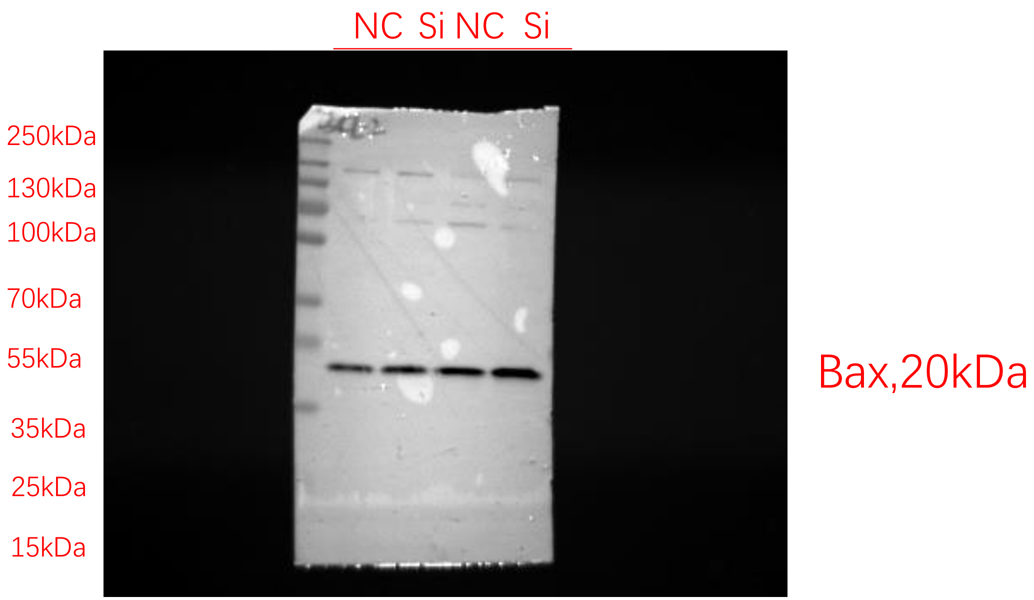

Third and Fourth

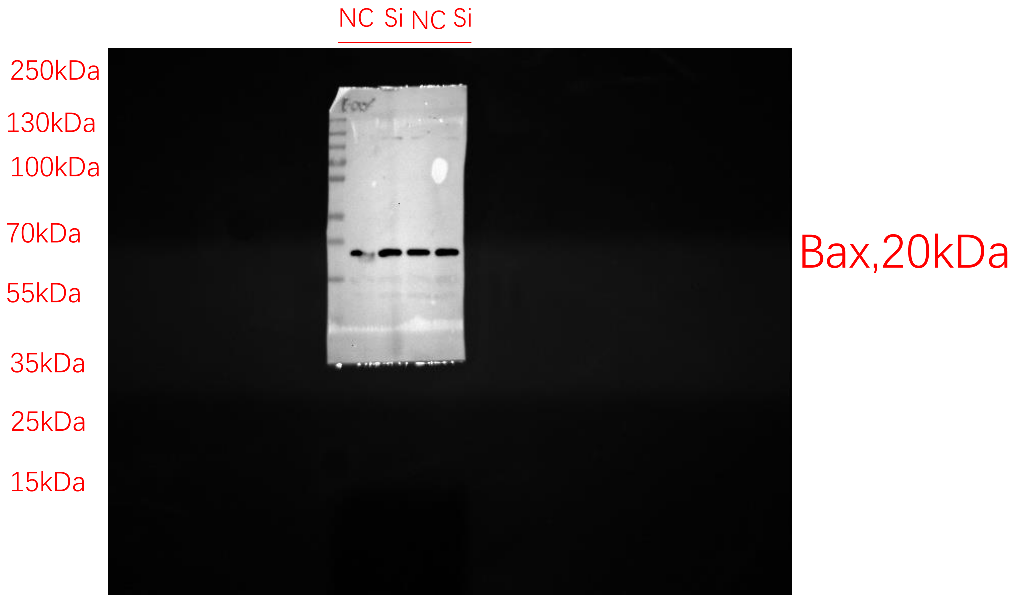

First and Second

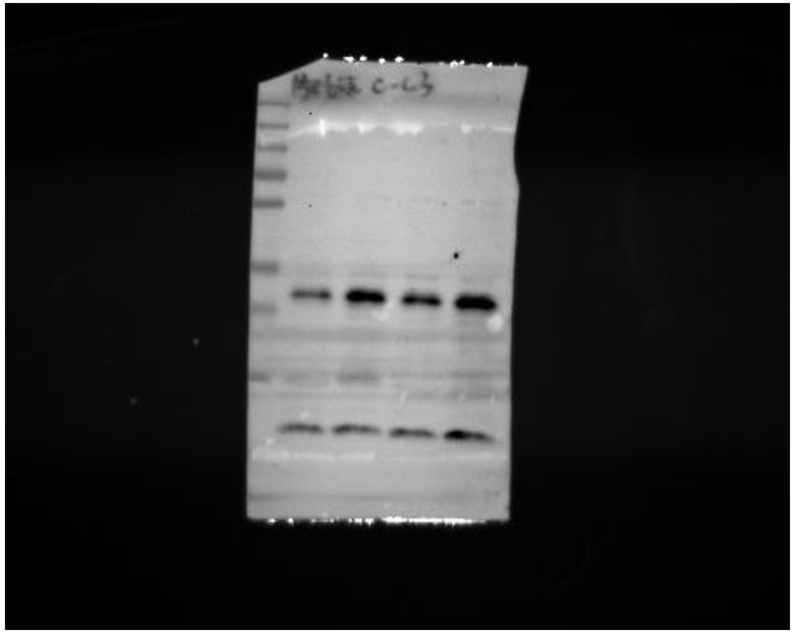

Third and Fourth

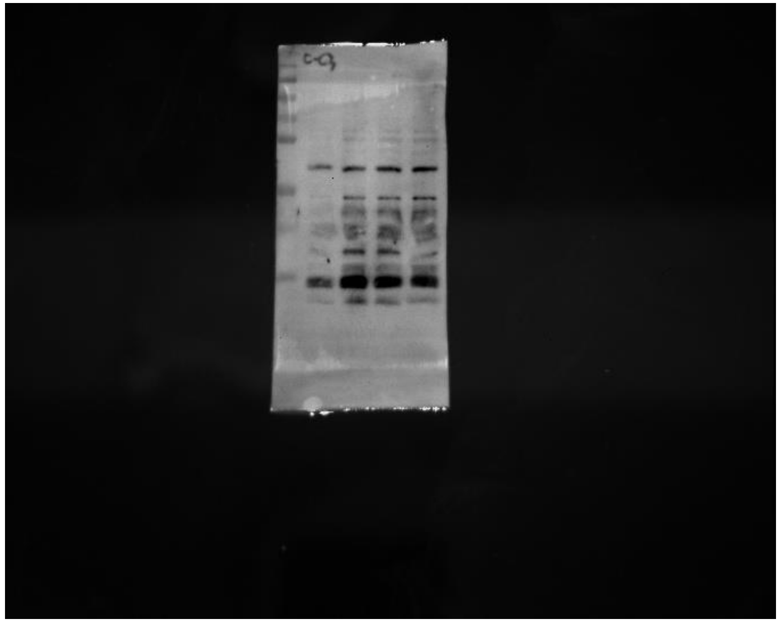

First and Second

NC Si NC Si

250kDa
130kDa
100kDa
70kDa
55kDa
35kDa
25kDa
15kDa

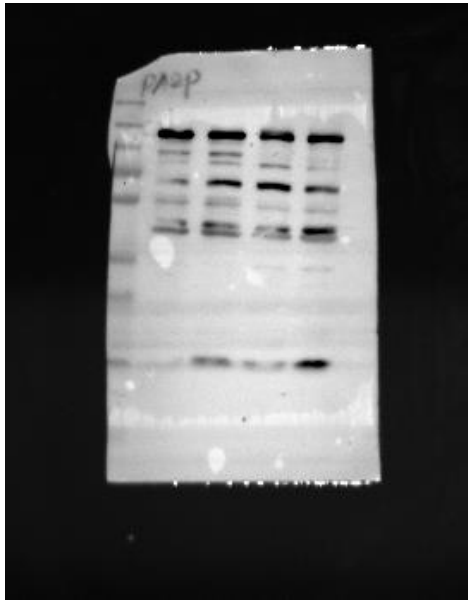

PARP 116kDa

Third and Fourth

NC Si NC Si

250kDa
130kDa
100kDa
70kDa
55kDa
35kDa
25kDa
15kDa

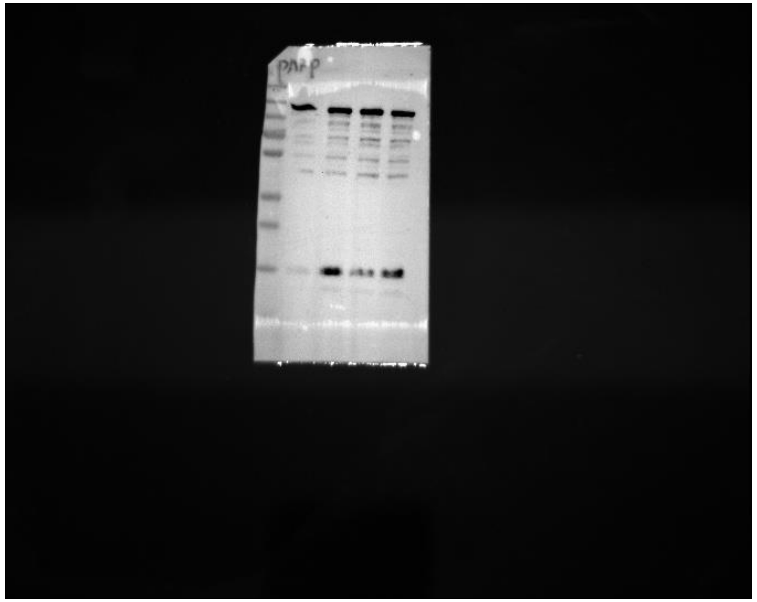

PARP 116kDa

First and Second

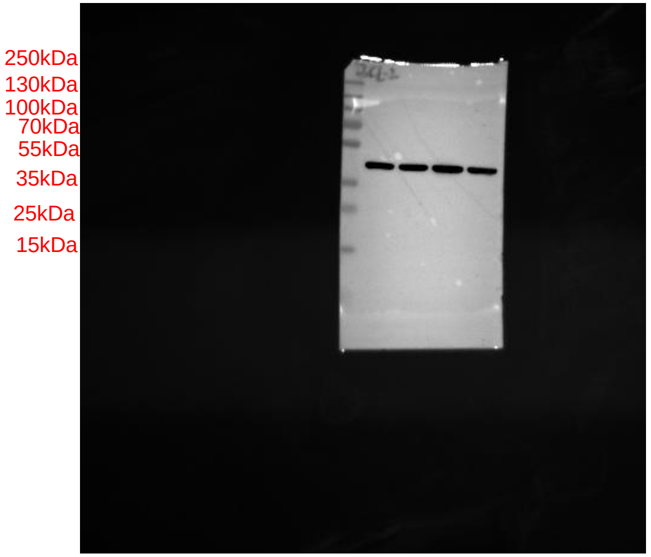

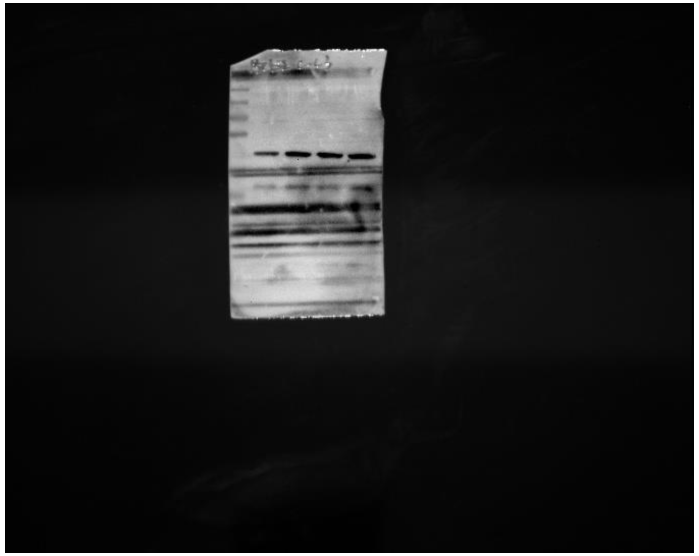

Third and Fourth

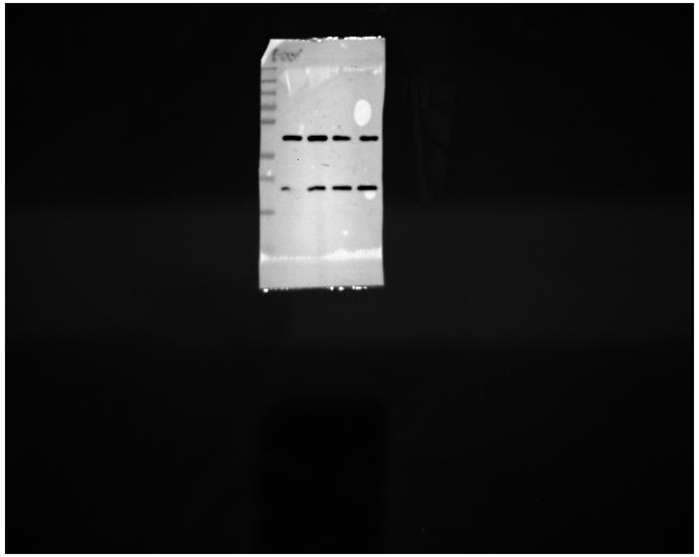

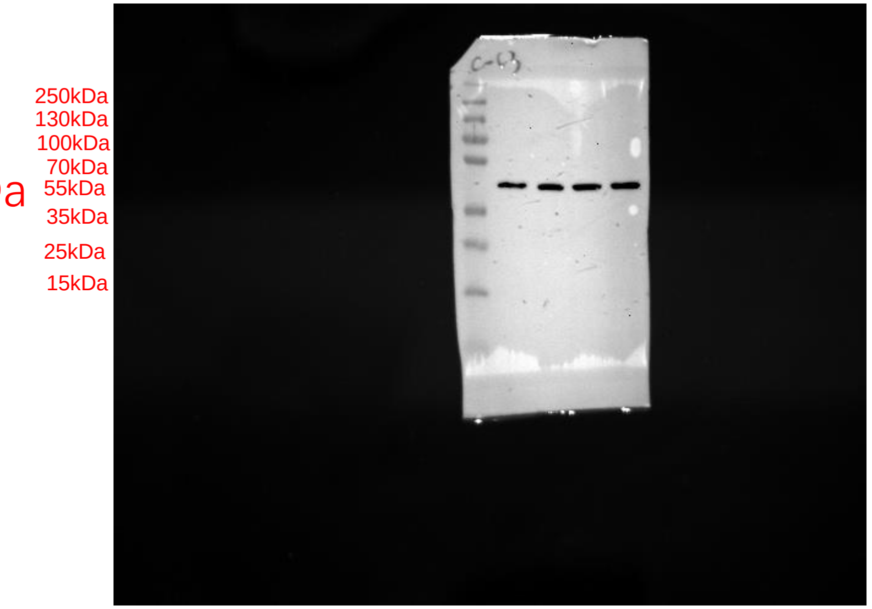

Supplement: Supplementary file 1 — Supplementary Material 1 [file 12885_2023_10713_MOESM1_ESM.pdf]
